# Discovery and cellular stress pathway analysis of 1,4-naphthoquinone derivatives with novel, highly potent broad-spectrum anticancer activity

**DOI:** 10.1186/s12929-018-0408-6

**Published:** 2018-02-08

**Authors:** Sajal K. Ghosh, Abhishek Ganta, Remco A. Spanjaard

**Affiliations:** 1 Cancer Center, Departments of Otolaryngology, Dermatology and Biochemistry, Boston University School of Medicine, 80 East Concord Street, Boston, MA 02118 USA; 2 Q-Ring, Inc, Brookline, MA 02446 USA

**Keywords:** Survivin, SAR, Therapy, Tumor, Plumbagin, ROS, Autophagy, ERK, Stress pathway, YM155

## Abstract

**Background:**

Chemotherapy and targeted therapies have made important strides in cancer treatment yet they often fail and new therapies are still needed. Here, we employed a phenotypic screen to identify and analyze the mechanism of action of novel small molecules that interfere with critical pathways involved in tumor cell growth, using chemoresistant A375 melanoma cells as a model.

**Methods:**

Cell culture studies were performed in ATCC-recommended media. Compounds, and compound libraries were obtained from Boston University or purchased commercially. Effects on A375 cell viability, proliferation and morphology were determined by Celigo Image Cytometer and viability staining. Anticancer activity of the lead compound was tested in a xenograft nude mouse model. Signaling and cell death pathways were analyzed by SDS-PAGE and immunoblotting, and/or fluorescence microscopy.

**Results:**

After evaluating 4477 compounds, one hit compound CB533 was identified that caused significant reduction of A375 cell growth. CB533 is an unexplored 1,4-naphthoquinone (NQ) derivative which unlike 1,4-NQ, induced rapid cell death without generating reactive oxygen species (ROS). Structure-activity relationship analysis showed that a pyrrolidine in the 1,4-NQ nucleus in lead compound Pyr-1 yielded optimal activity. CB533 and Pyr-1 had growth-suppressing effects on a large variety of chemotherapy-resistant cancer cell lines in the nano to picomolar range. Pyr-1 also significantly reduced growth of MDA-MB-231 breast cancer cells in nude mice. Pyr-1 rapidly induced activation of major stress pathways and autophagy, which was efficiently blocked by ERK, and somewhat by PI3K inhibitors.

**Conclusion:**

CB533 and lead Pyr-1 represent novel broad-spectrum, anticancer compounds that are up to 1000-fold more potent than plumbagin, a natural 1,4-NQ with known anticancer activity. Since the growth suppression activities of CB533 and Pyr-1 are unaffected by the chemotherapy resistance of cancer cells, these compounds have promising therapeutic potential. The pyrrolidine in the 3 position of the 1,4-NQ nucleus of Pyr-1 is a critical component of the pharmacophore. Pyr-1-induced cellular stress was mediated by an ERK, and to a lesser extent by an AKT-dependent pathway without involving apoptosis. Our data suggest that Pyr-1 derives its greatly enhanced antitumor activity via mimicking ROS-induced stress signaling without generating ROS, and likely committing cells to autophagy.

## Background

Cancer has historically been treated with chemotherapeutic agents that inhibit proliferation of all dividing cells, and consequently toxicity has always been a major problem. With increased understanding of molecular processes that drive progression of cancer, recent years have seen a shift in focus to targeted therapies that inhibit function of specific proteins that are thought to play critically important roles in tumor cell growth. This strategy has resulted in several new exciting drugs that to varying extent show improved efficacy, and more tolerable toxicity against a variety of cancer types [1]. Unfortunately, these drugs typically have a cytostatic rather than cytotoxic activity and often only a subset of patients with the correct genetic tumor profile can be treated. Moreover, redundancy or secondary mutations in the target commonly result in resistance to therapy after prolonged treatment. This is particularly true for late stage melanoma, a notoriously difficult to treat cancer type [2]. For these reasons there is renewed interest in cell-based, phenotypic screening methods that allow selection of compounds that cause desired outcomes such as growth-arrest, senescence or apoptosis. While the target initially remains unknown, and would have to be identified in subsequent studies, it is at least established early on in development that those compounds can modulate a clinically-relevant pathway [3–5].

As our starting model, we set out to identify novel small molecules with potent growth-suppressive activity against metastatic, chemoresistant A375 melanoma cells by employing a phenotypic screen that detects reduced cell numbers, cell viability and altered cell morphology as read-out for a successful hit. This strategy yielded CB533, a synthetic 1,4-naphthoquinone (1,4-NQ) derivative. 1,4-NQ forms the nucleus of many natural analogs such as vitamin K, which is important for e.g. blood coagulation, and plumbagin [6]. The latter compound is found in the roots of Chitrak (*Plumbago zeylanica*), a plant that has been used for centuries in Asian folk medicine against a multitude of ailments, and which has been shown to have numerous pharmacologic effects on a variety of cancer cells [7]. Structure-activity relationship (SAR) studies of CB533 resulted in optimized lead compound Pyr-1 which has significantly increased, broad-spectrum anticancer activity in both in vitro and in vivo cancer cell models. The activity of Pyr-1 appears to be specifically due to the presence of a pyrrolidine group in the pharmacophore. Finally, the signaling pathway and cell death-inducing mechanism of action of Pyr-1 was analyzed.

## Methods

### Phenotypic screening methods and compounds

Compound libraries: FDA Approved (640 compounds), CMLD-BU (1837 compounds) and ChemBridge Diversity Subset (2000 compounds) totaling 4477 compounds were obtained from Boston University, Boston, MA. A375 cells were plated in 384 well plates in 40 μl complete media until it was approximately 25% confluent. Compounds were added to wells at 1 μM final dose by Apricot Liquid Dispenser (Apricot Designs, Covina, CA), while control cells did not receive compound. After 4 days, cell numbers and morphology were analyzed by Celigo Image Cytometer (Nexcelom Bioscience, Lawrence, MA) and cell viability relative to control cells was determined after Alamar Blue viability staining (ThermoFisher Scientific, USA), by adding 4 μl to each well. After incubating plates for 1 h, viable cell numbers were determined by measuring absorbance at 570 nm by plate reader relative to untreated control. All compounds were either obtained from ChemBridge (Chembridge, San Diego, CA) or made in-house by the Department of Chemistry, Boston University, Boston, MA. Compounds were dissolved in DMSO but total DMSO concentration in cell culture experiments never exceeded 0.1%. Chemical names of most relevant compounds are, CB533: 2-(methylamino)-3-(1-piperidinyl)-1,4-naphthalenedione, Pyr-1: 2-(methylamino)-3-(1-pyrrolidinyl)-1,4-naphthalenedione.

### Cells, cell culture and compound-responsiveness studies

All cells were obtained from the American Type Culture Collection (ATCC) and cultured in media supplemented with serum according ATCC-recommended conditions in humidified atmosphere of 37 °C at 5% CO_2_. Cells were seeded at 5000 cells/well in 96 well plates. Experiments with the XenoSelect I panel listed in Table 2 were performed at least in triplicate by Crown Bioscience (San Diego, CA). Compounds were given over 9 dose ranges typically between 10^− 3^ and 10^2^ μM, plus DMSO control, for 72 h. As additional control, each tested cell line received one chemotherapeutic compound from the following group: doxorubicin, mitomycin or cisplatin at similar or higher dose (see Table 2). Cell viability was determined by MTS or CellTiter-Glo assay (Promega, Madison, WI). Surviving rate was calculated as % = (OD_test article_-OD_medium control_)/(OD_DMSO control_-OD_media control_)× 100%. IC_50_ of CB533, Pyr-1 and chemotherapeutic control were established where possible by fitting the curve using nonlinear regression with a sigmoidal dose response. The IC_50_ was automatically produced by GraphPad Prism 5.0. SD values were obtained for each triplicate data point and found to be < 10% of the mean.

### Animal studies

Animal studies were performed by Washington Biotechnology (Baltimore, MD). MDA-MB-231 cells were cultured according to ATCC-recommended conditions to 80% confluence, harvested and resuspended in serum-free media. Next, 4 × 10^6^ cells were injected via subcutaneous route in the right flank pad of female athymic nude mice. After 6 days, mice were randomly divided into 2 groups. 8 mice were injected intraperitoneally with 0.25 mg/kg Pyr-1 and 7 mice received DMSO solvent control with the same volume and concentration at a frequency of 5×/week. After 16 days the study was terminated due to tumors reaching maximum allowed size in the control group. Tumor volumes were measured 3 times weekly with calipers, and calculated using formula: length x width x width x ½. Toxicity was assessed as body weight loss (BWL). BWL was defined as the percentage of predosing body weight according to the formula**:** BWL = current weight of the body weight/predosing body weight × 100 -100. After euthanization, tumors were excised and their weight was measured. One-way ANOVA with Fisher’s LSD post hoc comparisons was used for statistica comparisons of tumor growth. *P* < 0.001 was considered significant. Student t-test was used for statistical comparison of excised Pyr-1-treated vs control tumor weight, where *p* < 0.05 was considered significant.

### Immunoblotting and quantitation

Cells were washed in PBS and harvested before disrupted in RIPA buffer (1% NP-40, 0.1% SDS, 50 mM Tris–HCl pH 7.4, 150 mM NaCl, 0.5% sodium deoxycholate, 1 mM EDTA) containing 5 μl/mL of combined protease and phosphatase inhibitor cocktail (Thermo Scientific). Protein samples (30 μg) were separated in readymade 4–20% MiniProtean–TGX precast polyacrylamide gels (BioRad, Hercules, CA) and transferred onto BA-85 nitrocellulose membranes (Perkin–Elmer, Boston, MA). Rabbit primary antibodies STAT3 (79D7), phosphoSTAT3-Tyr 705 (D3A7), Survivin (#2808), Caspase-3 (#9662), cleaved Caspase-3 (#9661) Bax (D2E11), Bad (9239), Bcl-2 (D55G8), pan-AKT (11E7), PhosphoAKT-308 (D25E6), PhosphoAKT-473 (D9E), pan-ERK(137F5), PhosphoERK-T202/Y204 (197G2), pan-p38 (#9212), Phospho-p-38 (#9215), pan-JNK/SAPK (#9252) and PhosphoJNK/SAPK (#9251) were all obtained from Cell Signaling Technology. Mouse monoclonal antibodies for VEGF (SC-7269) was from Santa Cruz Biotechnology (Santa Cruz, CA), whereas ß-actin (A1978) was from Sigma Chemicals. All primary antibodies were used at 1:1000 dilutions. Horseradish peroxidase-conjugated secondary antibodies were obtained from Amersham/GE Healthcare Corporation and used at 1:2000 dilutions. Bands were detected by Western Lightning Plus-ECL chemiluminescence substrates (Perkin-Elmer) and photographed in Imagequant LAS4000 (GE Healthcare).

To quantitate expression, band intensities were quantitated using Image Studio Lite software from LI-COR Biosciences (Lincoln, NE). Equal sized boxes were drawn around the bands of interest and density was obtained of the entire area. Background contribution was corrected by subtracting intensity of an identically-sized box of an unused area of the image from all bands of interest. To correct for protein loading, intensity of β-actin for all 3 conditions was determined in a similar manner and all protein expression measurements were normalized for β-actin levels in untreated cells. Next, protein expression level for each protein of interest was arbitrarily set at 1 in untreated cells, and relative levels for 1 and 4 h time points were calculated accordingly.

### ROS detection assay

To detect ROS, A375 cells were cultured in 6-well plates in 5 ml standard media described above till they reached 50-75% confluence. Cells then received fresh media that contained either 0.1% DMSO, 5 μM 1,4-NQ or CB533. After 3 h, all cells received 10 μl of a fresh solution of 2′,7′-Dichlorofluorescin diacetate dissolved in DMF (Abcam, Cambridge, MA) at final concentration of 10 mM. After 1 h, ROS was detected by green fluorescence using an Olympus IX51 bright field/fluorescence microscope equipped with a Fluorescein compatible filter (Ex/Em = 490/525 nm).

### Autophagy assay

Acidic autophagic vacuoles in A375 cells were detected by staining with acridine orange, as described previously [8] with modifications. Cells were grown in Nunc Lab-Tek II 4-well chamber slides (Fisher Scientific) and treated with Pyr-1 with or without inhibitors. Bafilomycin A1 (100 μM), PD98059 (50 μM) or LY294002 (50 μM) were added 1 h before Pyr-1 was added. Pyr-1 treatment (1.2 μM) was for 4 h. Acridine orange was added directly to the cells at 1 μg/ml final concentration for 20 min. Cells were then washed three times in PBS and mounted with Ultracruz mounting medium containing 4′,6-diamidino-2-phenylindole (DAPI, Santa Cruz Biotechnology) and immediately viewed under Olympus IX51 fluorescence microscope.

## Results

### Phenotypic screening identifies novel 1,4-NQ hit compound

**Fig. 1:**
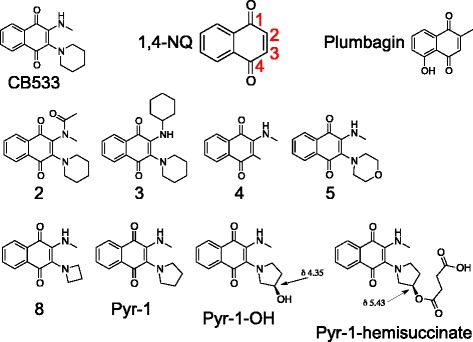
Structure and corresponding numbers of compounds listed in Table 1. Numbering of positions in the 1,4-NQ nucleus are shown in red

After the initial screening of 4477 compounds, 11 compounds were selected for further testing with multiple doses based on their cytotoxic effects and novelty in the scientific literature as tumor cell growth-suppressors. From this group, CB533 was identified as the hit compound with the strongest growth-suppressing activity against A375 cells. There were no indications for increased differentiation or senescence (data not shown). CB533 is a synthetic 1,4-naphthoquinone (1,4-NQ) derivative related to e.g. plumbagin, a highly-studied compound with known anticancer activity (Fig. 1) [6, 7]. To compare the activity of CB533 against these two molecules, A375 cells were plated and growth-suppressive activity of CB533, 1,4-NQ and plumbagin was tested in a dose-response assay with compound concentrations between 10 nM and 10 μM and 0.1% DMSO solvent control. After establishing the number of viable cells after 48 h, IC_50_ of CB533 was found to be 390 nM whereas that for 1,4-NQ and plumbagin, in agreement with earlier reports [6, 9], was in excess of 10 μM (Table 1). Thus, our data shows that CB533 is a novel 1,4-NQ compound with significantly increased activity over 1,4-NQ and plumbagin.

**Table 1:** IC_50_ of CB533 and derivatives obtained from A375 cells. See Fig. 1 for chemical structures

Compound	IC_50_ (nM)
CB533	390
1,4-NQ	> 10,000
Plumbagin	> 10,000
2	1500
3	> 10,000
4	> 10,000
5	3200
8	55
Pyr-1	10
Pyr-OH	270
Pyr-1-hemisuccinate	> > 500

### CB533 acts rapidly but does not generate ROS

**Fig. 2:**
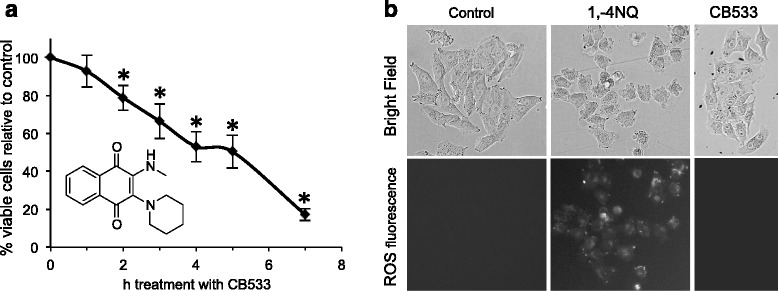
Kinetics of CB533 activity, and ROS assay in A375 cells. **a** Cells were treated with 5 μM CB533 and cell viability was determined at indicated time points. 4 ± 0.5 h after treatment number of viable cells was reduced by 50%. The experiment was performed three times, and a representative experiment is shown. Data are expressed as the mean ± SD of triplicate samples. *P* ≤ 0.05 compared to untreated control cells (by Student’s *t* test analysis). **b** Cells were treated for 4 h with 5 μM CB533, 1,4-NQ or DMSO control. Production of ROS is only observed in 1,4-NQ-treated cells. A representative experiment is shown

1,4-NQ compounds are generally regarded as redox recycling compounds that produce ROS, which in turn affects many signaling pathways leading to apoptosis and autophagy [10–14]. To test whether CB533 would have a similar mode of action, we first established the kinetics of CB533-induced growth-suppression. As shown in Fig. 2a, 50% of A375 cells were no longer viable after 4 ± 0.5 h treatment. To test whether this effect was associated with ROS production, A375 cells were again treated for 4 h with DMSO control, 1,4-NQ or CB533 and ROS was detected by fluorescence microscopy. As shown in Fig. 2b, as expected 1,4-NQ induced clearly detectable levels of ROS but surprisingly, CB533-treated cells did not show observable ROS generation, and remained indistinguishable from control cells.

### SAR analysis identifies critical functional group

The previous results suggest that the functional groups of CB533 in the 2 and/or 3 positions of the 1,4-NQ nucleus [6] are responsible for the increased activity of CB533 over 1,4-NQ and plumbagin. To test this hypothesis, a limited number of derivatives with altered functional groups in these positions were assessed for their dose-dependent effects on A375 cell growth, and IC_50_ values were again obtained after viability staining (see Fig. 1 and Table 1). Substitution of the methylamino group in position 2 by groups with increasing bulk in compounds 2 and 3 resulted in increasing IC_50_ values at 1.5 and > 10 μM, respectively. Next, the importance of position 3 was evaluated. Here, reducing group size by removal of the piperidine in compound 4 greatly increased IC_50_ to > 10 μM suggesting that this ring size and/or structure may be important. In agreement with this interpretation, substitution of piperidine by morpholine, which maintains ring size but with introduction of an oxygen alters effects on pKa and electron release into the 1,4-NQ core, also increased IC_50_ by about 10 fold to 3.2 μM. We then scanned effect of ring size on activity by replacing the piperidine by azetidine in compound 8, and pyrrolidine in Pyr-1. Interestingly, this resulted in much improved activity with IC_50_ for compound 8 at 55 nM and 10 nM for Pyr-1, which is about 1000 fold more potent than 1,4-NQ or plumbagin [6], These data show that the pyrrolidine ring size in lead compound Pyr-1 is of optimal size and composition.

Finally, since Pyr-1 and other 1,4-NQ derivatives have poor water solubility (data not shown) which makes potential formulation more complex, we wanted to test whether this property could be improved. For this purpose, the pyrrolidine in Pyr-1 was modified by adding a hydroxyl group in Pyr-1-OH and a hemisuccinate in Pyr-1-hemisuccinat. However, we again found that any change in the pyrrolidine ring structure resulted in significantly reduced activity. These combined SAR studies suggest that for optimal activity, the 2 position should contain a small group, but specifically in the 3 position of the 1,4-NQ nucleus an unmodified pyrrolidine is a critical component of the Pyr-1 pharmacophore.

### CB533 and Pyr-1 suppress growth of a wide range of cancer cells

To test whether CB533 and Pyr-1 would also have growth-suppressing activity on other cancer cell types, dose-response growth curves were obtained on a large cell panel representing breast, ovarian, colon, prostate, lung, pancreas, liver, head and neck, kidney and stomach cancer in addition to sarcoma, melanoma, glioma, leukemia and myeloma. A375 cells were included as compound response reference control. Additionally, chemotherapeutic agents doxorubicin, cisplatin or mitomycin C were added to compare activity of CB533 and Pyr-1 to clinically-established therapeutics. IC_50_ was determined for each assay and results are listed in Table 2. As before, similar results for CB533 and Pyr-1 on A375 cells were detected with IC_50_ of 334.5 and 14.5 nM, respectively. This is notably more potent than the effects of cisplatin with IC_50_ of 7.1 μM. Over all cell lines tested, CB533 showed IC_50_ results ranging from 5.4 nM for highly sensitive SK-BR-3 cells to 2.144 μM for more resistant U-87 MG glioma cells. Note again that SK-BR3 cells are highly resistant to cisplatin, with IC_50_ of almost 100 μM. Curiously, for Pyr-1 the most sensitive and resistant cells were ovarian cancer cells: OV-CAR-3 cells had an IC_50_ of 0.6 nM whereas that for SK-OV-3 cells was 60.1 nM. Relative to chemotherapeutic compounds, Pyr-1 was much more potent in these cells with IC_50_ for cisplatin and mitomycin C of 6.31 μM and 1.06 μM, respectively. The average IC_50_ for hit compound CB533 calculated with data from 39 cell lines was 401.3 ± 495.5 nM, whereas this number significantly improved for lead compound Pyr-1 to 14 ± 16.7 nM compiled over 40 cell lines. Thus, CB533 and in particular Pyr-1 showed broad-spectrum cancer growth-suppressive activity in the low to very low nM to even pM range, often in cases where conventional therapeutics essentially had no effects. These results suggest that CB533 and Pyr-1 act though a different mechanism that can bypass chemotherapy resistance.

**Table 2:** IC_50_ of CB533, Pyr-1 and chemotherapeutic agents (Control) obtained from a panel of cancer cells

Tissue	Pyr-1, IC_50_ (nM)	CB533, IC_50_ (nM)	Control, IC_50_ (nM)
Breast			
BT4740	2.1	5.7	Dox: > 10^5^
MCF-7	2.8	72.8	Cis: 14,800
SK-BR-3	1	5.4	Cis: 99,410
Colon			
CoLo205	3.2	42.9	Mit: 2610
DLD-1	10.1	227	Cis: 4680
HCT116	1.6	163.5	Mit: 4290
HT29	4.7	170.3	Cis: 10,570
SW480	1.4	88.4	Cis: ≈10^5^
SW620	0.9	55.3	Cis: > 10^5^
Prostate			
LNCaP	18	1540	Cis: 13,520
DU145	19	405.8	Cis: 1680
PC-3	5.9	76.8	Cis: 18,270
Pancreas			
BxPC-3	4.5	108.8	Cis: 5550
PANC-1	1.2	267	Cis: 4300
MIA PaCa-2	16.9	283.9	Cis: 13,540
Lung			
A549	9.9	36.9	Cis: 11,740
CaLu-6	14.4	242.3	Cis: 2380
NCI-H460	6.9	714.6	Cis: 780
NCI-H1703	19.5	nt	Cis: 13,670
SK-MES-1	12.8	382.5	Cis: 14,480
Myeloma			
RPMI-8226	12.6	59	Cis: 11,010
Ovary			
SK-OV-3	60.1	666.6	Mit: 1060
OVCAR-3	0.6	46.8	Cis: 6310
Liver			
Hep 3B	76.2	197	Cis: 6950
Hep G2	3.5	46.5	Mit: 2700
SK-HEP-1	48	1454	Mit: 3500
Stomach			
BGC-823	10.5	172.5	Cis: 1250
MCG803	3.3	179.3	Cis: 6550
NCI-N87	2.2	310.3	Cis: 5830
Head and Neck			
KB	11.2	120.4	Cis: 12,340
CNE2	8.4	971.6	Cis: 11,820
Kidney			
786-O	39.5	986.4	Cis: 4280
Sarcoma			
HT-1080	12.9	663.3	Dox: 320
143B	19.1	1360	Mit: 1480
Melanoma			
A375	14.3	334.5	Cis: 7100
SK-MEL-5	13.3	245.5	Cis: 5130
A2058	1.7	511.5	Cis: 7240
Glioma			
U-87 MG	40	2144	Cis: 4480
Leukemia			
K-562	7.4	99.6	Cis: 2700

*Dox* doxorubicin, *Mit* mitomycin *C*, *Cis* Cisplatin

### Pyr-1 has significant antitumor effects in nude mouse breast cancer model

**Fig. 3:**
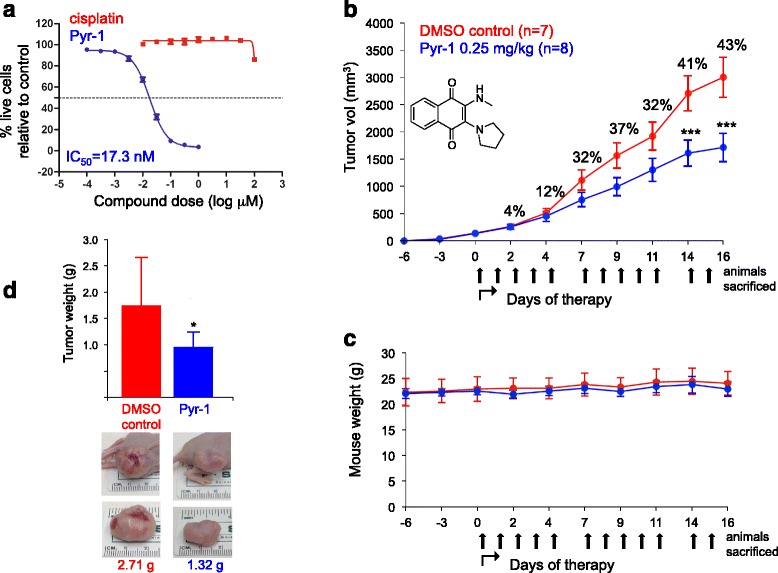
Pyr-1 significantly suppresses growth of MDA-MB-231 breast cancer cells in cell culture and nude mouse models. Clockwise: **a** Cells were treated with Pyr-1 or cisplatin control at indicated dose, and IC_50_ was determined after 48 h by viability staining. **b** Nude mice were implanted with tumor cells, and therapy with Pyr-1 (blue) or DMSO solvent control (red) was initiated 6 days later. Average tumor size reduction in Pyr-1-treated versus control group is shown in percentiles. **, *p* < 0.001 (by one-way ANOVA). **c** Mouse body weight was recorded to monitor potential treatment-associated toxicity. **d** 16 days after treatment, study was terminated, tumors were excised and average weight was determined. *, *p* < 0.05 compared to DMSO control (by Student’s *t* test analysis)

As shown in Fig. 3a and Table 2, Pyr-1 showed excellent activity against triple negative MDA-MB-231 breast cancer cells that are highly resistant to cisplatin (IC_50_ = 17.3 nM and > 100 μM, resp). To establish whether Pyr-1 can be used as a potential therapeutic in vivo, we chose this model as a representative, extremely difficult to treat cancer type. Nude mice were injected sc with MDA-MB-231 cells. After 6 days, mice were divided into 2 groups that either received 5 ip injections/week of 0.25 mg/kg Pyr-1 or solvent control. Mouse weight was monitored for potential Pyr-1-associated toxicity, and tumor size was determined 3×/week. After 16 days experiments were terminated when tumors in the control group reached maximum allowed size. As shown in Fig. 3b, in the Pyr-1-treatment group tumors already began to show slower growth at day 2 of therapy. This effect became more pronounced over time, with tumor sizes in the Pyr-1-treatment group growing to 32% smaller size after 7 days of treatment. Highly statistically significant differences were observed after days 14 and 16, where tumors in the Pyr-1 therapy group were 41% and 43% smaller than in the control group, respectively. No signs of toxicity in the treatment groups were observed (Fig. 3c). Finally, at day 16 tumors were also excised and weighed. As shown in Fig. 3d, on average, control tumors weighed 1.74 g, whereas Pyr-1-treated tumors weighed 0.95 g, showing that Pyr-1 therapy reduced tumor weight by about 45%, in agreement with the in situ measurements.

### Pathway analysis of Pyr-1-induced signaling

**Fig. 4:**
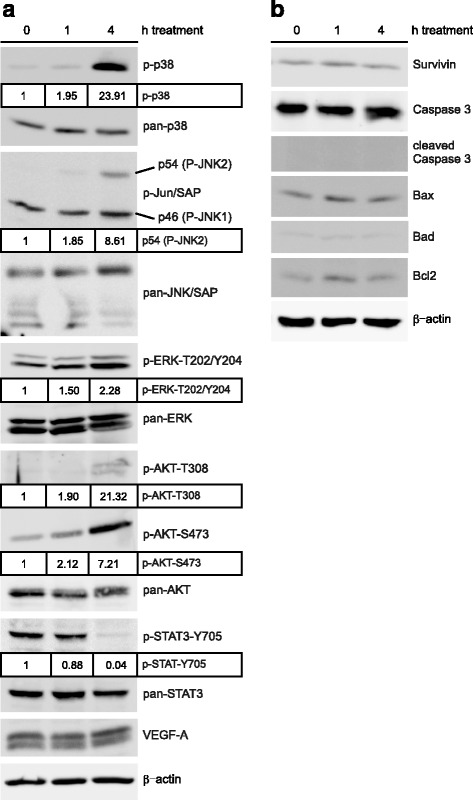
Immunoblot analysis of induction of stress (**a**) and/or apoptotic cell death (**b**) pathways in A375 cells by treatment with Pyr-1 at indicated time points. β-actin expression served as loading control. Quantitation of differential protein expression levels in treated relative to untreated control cells (arbitrarily set at 1) are indicated in boxes where observed

1,4-NQ derivatives such as plumbagin have been extensively investigated for their effects on stress and survival pathways. To analyze these pathways, A375 cells were treated for 0 (control), 1 h or 4 h with Pyr-1 and whole cell lysates were prepared. Next, proteins were separated by SDS-PAGE and expression and quantitation of critical proteins was established by immunoblot and band intensity analysis, respectively. As shown in Fig. 4a, robust phosphorylation and activation of p38, p54/JNK2 (but not p46/JNK1), AKT-T308, AKT-S473 and to a lesser extent ERK1,2-T202/Y204, could be detected primarily after 4 h treatment, while overall expression levels remained unchanged. In contrast, STAT3-Y705 expression was strongly downregulated without altering total STAT3 levels. Expression of VEGF-A, reportedly down-regulated by plumbagin in myeloid leukemia cells [15], remained unchanged. These results establish that major stress pathways are activated by Pyr-1 that have been linked to cell death mechanisms. To analyze a possible role for Pyr-1 in apoptosis, as previously reported for plumbagin [7, 9, 12–14, 16–23] and survivin-suppressing analog YM155 [11], the effects of Pyr-1 on major cell death regulators was studied. As shown in Fig. 4b, Pyr-1 did not induce Caspase 3 activation, nor were there changes in expression of Survivin, Bcl-2, Bax and Bad. No significant apoptosis was detectable by FACS or fluorescence microscopy analysis either (data not shown).

### Pyr-1 induces ERK-dependent autophagy

**Fig. 5:**
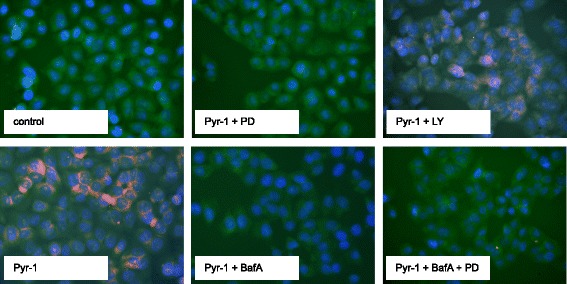
Pyr-1 (4 h treatment) induced autophagy in A375 cells as shown by acridine orange staining which could be completely blocked by bafilomycin A1 (BafA), PD98059 (PD), or PD + LY294002 (LY), but only partially blocked by LY. No autophagy was seen in control-treated cells

1,4-NQ derivatives plumbagin and YM155, a survivin-suppressing anticancer drug that is being evaluated in clinical trials, often show apoptotic or dual apoptosis and autophagy-mediated cell death. To test whether Pyr-1 induced autophagy, A375 cells were treated for 4 h with Pyr-1, in the absence of or presence of bafilomycin A1 (autophagy inhibitor that blocks fusion between autophagosomes and lysosomes), PD98059 (MEK1/MAP2K/ERK inhibitor) and/or LY294002 (PI3K inhibitor). Cells were stained with the lysotrophic dye acridine orange to detect acidic autophagic vacuoles and examined by fluorescence microscopy. The results in Fig. 5 provide evidence for autophagy in the Pyr-1-treated cells which was completely inhibited by bafilomycin A1. Interestingly, autophagy was also very effectively blocked by PD98059, but only partially by LY294002. The combination of bafilomycin A1 and PD98059 again completely inhibited appearance of acridine orange-stained cells. These results suggest that Pyr-1-induced autophagy, but not apoptosis underlies its growth-suppressing activity, and that this effect is dependent on activated ERK. However, activated AKT also contributes to some extent to this process.

## Discussion

Phenotypic screening for novel compounds with growth-suppressive activity against chemoresistant A375 cells yielded unexplored, synthetic hit compound CB533, a molecule which shares its 1,4-NQ nucleus with natural compounds with well-known anticancer activity such as plumbagin [6, 9]. Unlike plumbagin, however, CB533 did not produce ROS, and it had much stronger growth-suppressing activity. Subsequent SAR studies revealed in particular the critical role of pyrrolidine in the 3 position of the 1,4-NQ nucleus. We showed that this was the optimal ring size, and that any alterations to the ring decreased its activity. Lead compound Pyr-1 displayed approximately 1000-fold increased activity over plumbagin, and has vastly superior activity over other plumbagin-based natural and synthetic analogs [7, 10]. Pyr-1 suppressed growth of every tested cancer cell line with IC_50_ in the low nM range, or even pM levels in case of SW620 colon and OVCAR-3 ovarian cancer cells. The vast majority of these cell lines were highly resistant to standard chemotherapeutics suggesting that CB533 and Pyr-1 act through a different, conserved mechanism that is independent of clinical biomarkers such as p53, Her2/neu, or estrogen receptor status. The promising therapeutic potential of Pyr-1 was confirmed in a nude mouse xenograft model for triple-negative breast cancer, an extremely difficult to treat type of breast cancer with few viable therapeutic options. Treatment with Pyr-1 reduced tumor size by almost half compared to control tumors. It is conceivable that these results could be improved upon with additional fine-tuning of the drug regimen. For instance, YM155, a 1,4-NQ containing small molecule that was selected for its ability to suppress expression of the anti-apoptosis gene Survivin and which has very similar potency as Pyr-1 in both cellular and animal studies, was much more efficacious when delivered continuously through a pump rather than by injections [11, 24–26]. Pyr-1, which has a similar half-life as YM155 (data not shown), could conceivably show similar improvements in therapeutic outcome.

One factor that may contribute to the enhanced activity of Pyr-1 may be related to its pharmacophore and putative binding target. We hypothesize that Pyr-1, via its pyrrolidine group, binds to an as yet unknown critical mediator(s) of ROS signaling, thereby activating this pathway in the absence of ROS. This concept is conceivable, since YM155 is a specific target for Interleukin Enhancer-binding Factor 3/NF110 [27], and docking simulation studies have shown potential binding sites for plumbagin in PI3Kγ, AKT1/PKBα, Bcl-2, NFκB and STAT3, thereby interfering with their activity [28]. In that regard, it would be very interesting to see whether the pyrrolidine identified here increases activity of other 1,4-NQ derivatives, and secondly, whether it has to be in the same position identified here, or whether it could also function in other positions. These questions cannot be answered here, but will have to be addressed in future studies.

The signaling pathway activated by Pyr-1 proved to be very interesting. Considering the chemical similarities yet profoundly differential levels of potency and produced ROS by plumbagin and Pyr-1, we hypothesized that they would modulate activity of both common as well as different critical stress pathway mediators. Indeed, Pyr-1-induced activation of p38, pJNK/SAP and down-regulation of STAT3 which has also been reported for plumbagin-treated cells [7, 13, 14, 23, 29]. In contrast to plumbagin, however, Pyr-1 did not suppress expression of survivin [14], VEGF-A [15, 23] and Bcl-2 [7, 14, 15], did not activate Caspase 3 [7, 14], and induced activation, rather than suppression, of ERK1/2 [30, 31] and AKT [7, 8, 14, 20, 22]. The involvement of ERK and AKT may be of particular importance for the specific cytotoxic activity of Pyr-1. PI3K activates AKT in response to stress signals, which in turn can inhibit Caspase 3 activation and prevent apoptosis, as also seen for Pyr-1. It is thought that through a set of still not fully-understood signals, suppression of apoptosis can shift the cell death mechanism equilibrium towards autophagy, and vice versa [32, 33]. Activation of p38, JNK2, ERK and suppression of phosphorylated STAT3 have already been associated with autophagy [34–37] and all of these pathways are simultaneously engaged by Pyr-1. We speculate that activation of AKT, by turning off apoptosis and redirecting even more of the cell’s resources towards autophagy, may increase the efficiency and kinetics of cell death compared to e.g. plumbagin and YM155, which appear to have dual and thus possibly internally competing apoptosis and autophagy-inducing mechanisms [8, 38]. The precise nature of the autophagy-inducing mechanism of Pyr-1 and its (quantitative) effects on autophagic flux relative to plumbagin and YM155 will be the subject of future studies.

## Conclusion

The results from our phenotypic screen confirmed the value of this drug discovery strategy. Our research suggests that the surprisingly high potency of Pyr-1 is due to its ability to mimic activation by ROS, thereby activating major stress pathways that likely drive cells to autophagy. The antitumor activity of Pyr-1 was observed in all tested chemotherapy resistant cancer cells with IC_50_ in the low nM to pM range. According to the guidelines of the Drug Evaluation Branch (at the National Cancer Institute), Pyr-1 was anti-tumorigenic after 16 days of therapy. Pyr-1 may represent a new class of ROS-signaling mimetics that trigger a ROS response pathway without generating detectable levels of ROS, and as such is a potential new member of the emerging category of autophagy-modulating cancer therapeutics [39–42].

## Abbreviations

1,4-NQ: 1,4-Naphthoquinone
AKT: Protein kinase B
ATCC: American type culture collection
BafA: Bafilomycin A1
Bcl-2: B cell lymphoma 2
BWL: Body weight loss
Cis: Cisplatin
DMF: Dimethyl formamide
DMSO: Dimethyl sulfoxide
Dox: Doxorubicin
ERK1/2: Extracellular signal–regulated kinase 1/2
FACS: Fluorescence-activated cell sorting
IC_50_: Half maximal inhibitory concentration
JNK: c-Jun-N-terminal kinase
MAPK: Mitogen-Activated Protein Kinase
Mit: Mitomycin C
MTS: 3-(4,5-dimethylthiazol-2-yl)-5-(3-carboxymethoxyphenyl)-2-(4-sulfophenyl)-2H-tetrazolium)
NFκB: Nuclear factor kappa-light-chain-enhancer of activated B cells
PI3K: Phosphatidylinositol-4,5-bisphosphate 3-kinase
ROS: Reactive oxygen species
SDS-PAGE: Sodium dodecyl sulfate-polyacrylamide gel electrophoresis
STAT3: Signal transducer and activator of transcription 3
VEGF-A: Vascular endothelial growth factor A
